# Recent Advances of Interventional Endoscopic Retrograde Cholangiopancreatography and Endoscopic Ultrasound for Patients with Surgically Altered Anatomy

**DOI:** 10.3390/jcm10081624

**Published:** 2021-04-12

**Authors:** Yuki Tanisaka, Masafumi Mizuide, Akashi Fujita, Tomoya Ogawa, Masahiro Suzuki, Hiromune Katsuda, Youichi Saito, Kazuya Miyaguchi, Tomoaki Tashima, Yumi Mashimo, Shomei Ryozawa

**Affiliations:** Department of Gastroenterology, Saitama Medical University International Medical Center, 1397-1, Yamane, Hidaka, Saitama 350-1298, Japan; mizuide1971@yahoo.co.jp (M.M.); a.fujita0628@gmail.com (A.F.); t.ogawa0210@icloud.com (T.O.); msuzgast@tmd.ac.jp (M.S.); hk0112@saitama-med.ac.jp (H.K.); stm_ys41@yahoo.co.jp (Y.S.); kaz.hr77@gmail.com (K.M.); tomo3029@saitama-med.ac.jp (T.T.); ymashimo@saitama-med.ac.jp (Y.M.); ryozawa@saitama-med.ac.jp (S.R.)

**Keywords:** endoscopic retrograde cholangiopancreatography, altered anatomy, ERCP, balloon enteroscope, single balloon enteroscopy, double balloon enteroscopy, endoscopic ultrasound, EUS, interventional EUS, EUS-BD

## Abstract

Endoscopic retrograde cholangiopancreatography (ERCP) is considered to be the gold standard for diagnosis and interventions in biliopancreatic diseases. However, ERCP in patients with surgically altered anatomy (SAA) appears to be more difficult compared to cases with normal anatomy. Since the production of a balloon enteroscope (BE) for small intestine disorders, BE had also been used for biliopancreatic diseases in patients with SAA. Since the development of BE-assisted ERCP, the outcomes of procedures, such as stone extraction or drainage, have been reported as favorable. Recently, an interventional endoscopic ultrasound (EUS), such as EUS-guided biliary drainage (EUS-BD), has been developed and is available mainly for patients with difficult cases of ERCP. It is a good option for patients with SAA. The effectiveness of interventional EUS for patients with SAA has been reported. Both BE-assisted ERCP and interventional EUS have advantages and disadvantages. The choice of procedure should be individualized to the patient’s condition or the expertise of the endoscopists. The aim of this review article is to discuss recent advances in interventional ERCP and EUS for patients with SAA.

## 1. Introduction

There is a large variety of biliary tract diseases, such as bile duct stones and benign/malignant biliary strictures. They lead to hepatobiliary dysfunction, cholangitis, and eventually liver failure requiring appropriate therapy. Since its introduction in 1968, endoscopic retrograde cholangiopancreatography (ERCP) is thought to be the gold standard for diagnosis and interventions in biliopancreatic diseases. It has been reported that ERCP-related procedures have achieved success in approximately 95% of cases [1,2]. However, it is technically challenging to perform ERCP in patients with surgically altered anatomy (SAA), such as Roux-en-Y gastrectomy, hepaticojejunostomy with Roux-en-Y, pancreaticoduodenectomy, or Billroth II gastrectomy. First of all, the afferent limb, increased intestinal curvature, or postoperative adhesions hinder accessibility of the target site, such as the papilla or the hepatico/pancreatojejunal anastomosis. Next, selective biliary cannulation and subsequent procedures, such as stone extraction or drainage, are more difficult in patients with SAA than cases with normal anatomy. Outcomes using a conventional duodenoscope have not been satisfactory [3,4]. Hence, alternative treatments, such as percutaneous transhepatic biliary drainage (PTBD), have been widely applied to patients with SAA [5,6]. One study from a tertiary referral endoscopy center reported that the afferent loop intubation and cannulation success rates using side-viewing duodenoscope in patients with Billroth II gastrectomy were 86.7% (618/713 patients) and 93.8% (580/613 patients). The main reason for intubation failure was a long and angulated afferent loop [7]. Another systematic review and meta-analysis reported that the afferent loop intubation and cannulation success rates using a forward-viewing endoscope in patients with Billroth II gastrectomy were 91.1% and 92.3%. The subgroup analysis of the forward-viewing endoscope showed that the success rates of afferent loop intubation using the forward-viewing endoscope with cap-fitting (92.5%) was higher than the forward-viewing endoscope without cap-fitting (88.6%). The success rates of cannulation using the forward-viewing endoscope with cap-fitting (93.7%) was higher than the forward-viewing endoscope without cap-fitting (89.2%) [8]. These studies showed the usefulness of a conventional side or forward-viewing scope in patients with Billroth II gastrectomy. However, these scopes cannot achieve the afferent loop intubation in 10% of patients due to a long and angulated afferent loop.

Since the introduction of the balloon enteroscope (BE) for small bowel disorders [9], balloon-assisted ERCP, such as single-balloon enteroscopy (SBE)-assisted ERCP, or double-balloon enteroscopy (DBE)-assisted ERCP, have been developed for patients with SAA. Despite the evident effectiveness of BE-assisted ERCP, it is still more challenging to perform than ERCP in patients with normal anatomy in terms of scope insertion, biliary cannulation, and subsequent diagnostic and interventional procedures, such as forceps biopsy, stone extraction, and stent placement. Recently, interventional endoscopic ultrasound (EUS), such as EUS-guided biliary drainage (EUS-BD) or EUS-guided antegrade intervention, have been available for difficult cases of ERCP, making it a good option for patients with SAA. In this review, we discuss recent advances in interventional ERCP and EUS for patients with SAA.

## 2. Balloon Enteroscope

**Figure 1:**
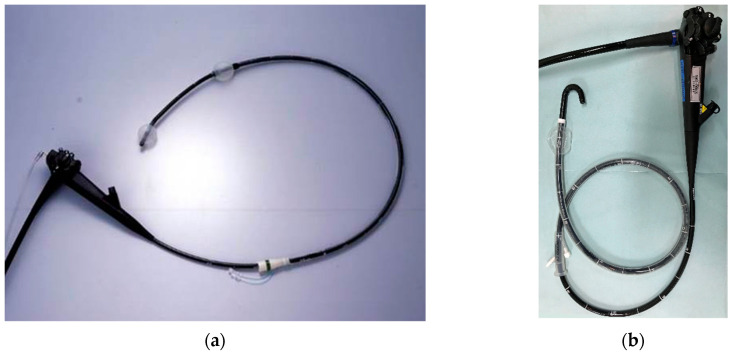
Balloon enteroscope: (**a**) double-balloon enteroscopy and (**b**) single-balloon enteroscopy.

Table 1 shows the specifications of the SBE and DBE presently available. The BEs are advanced by holding and shortening the intestine with an inflated balloon. The difference of SBE and DBE is the number of balloons (Figure 1). A balloon is attached to the tip of the over-tube for SBE. DBE equips two balloons. One is attached to the tip of the endoscope while another is attached to the tip of the over-tube. Moreover, the working channel port in SBE appears in an 8 o’clock direction on the endoscopic screen. In contrast, it shows in a 5:30 o’clock direction for DBE.

Use of conventional SBE and DBE is limited by their long working length of 200 cm. Therefore, only a few ERCP accessories are available. Recently, a short-type SBE (short SBE) and DBE (short DBE) with a working length of 152 cm (short SBE) and 155 cm (short DBE), and with a working channel diameter of 3.2 mm is available to increase accessories that can be used for BE-assisted ERCP. Moreover, the short SBE permits the function of passive bending and high-force transmission [10], and the short DBE permits the function of adaptive bending and advanced force transmission [11]. When using SBE, if the scope is at the intestinal tract wall when passing through a sharp flexure, then the passive bending section allows the scope to smoothly bend along the bend of the wall, making it possible to move forward. High-force transmission capabilities make it possible to perform torque operations efficiently and to provide better scope control. Therefore, it is also useful for bile duct cannulation and subsequent treatment procedures. In short, DBE, adaptive bending, and advanced force transmission provide a similar role to passive bending and high-force transmission. These features have contributed to overcoming the difficulties of scope insertion to the target site or biliary cannulation.

In general, ERCP-related procedures using BE are performed under conscious sedation, such as intravenous midazolam and pethidine. During scope insertion, patients are positioned in the prone position. However, for difficult cases, the position may be changed or abdominal pressure may be used. In case the BE forms a loop during insertion, the small intestine is fixed using the inflated balloon and shortened by withdrawing the BE. It is useful and safe for scope insertion to use carbon dioxide. In some difficult cases, such as long afferent limbs seen in Roux-en-Y reconstruction cases, it is difficult to proceed to the target site using short BE. Hence, a change to a conventional-type enteroscope (working length of 200 cm) is required [12]. A transparent hood is useful not only for scope insertion but also for subsequent procedures, such as biliary cannulation [13]. Since postoperative adhesions tend to occur in patients with SAA, endoscopists could feel adhesions during scope insertion or shortening. It must be taken into consideration that there is an increased risk of perforation during scope insertion in patients with SAA than in anatomically normal cases. After achievement of scope insertion to the target site, biliary cannulation is performed using a catheter with a guidewire for cholangiography and deep cannulation. After biliary cannulation, endoscopic diagnosis/interventions, such as stone extraction, stent placement, and biopsy/cytology for diagnosis are performed.

Although endoscopic sphincterotomy (EST) is one of the common procedures in ERCP, it can be particularly troublesome in patients with SAA (Billroth II gastrectomy or Roux-en-Y gastrectomy). It is considered to be difficult because the correct direction of the incision is sometimes uncertain due to the upside-down position in these patients. If the incision is made in the wrong direction, perforation could occur. One study from a tertiary referral endoscopy center evaluated 40 cases of the endoscopic papillary large balloon (over 10-mm) dilation (EPLBD) without EST for stone extraction in patients with Billroth II gastrectomy. Stones were successfully removed in all cases. Acute complications from EPLBD included mild pancreatitis in two patients (5.0%) [14]. This result showed the usefulness and safety of EPLBD without EST. If an endoscopist feels difficult to perform EST in patients with Billroth II gastrectomy or Roux-en-Y gastrectomy, EPLBD without EST may be recommended.

## 3. Single Balloon-Assisted ERCP

Table 2 shows outcomes of SBE-assisted ERCP procedures in patients with SAA [12,13,15,16,17,18,19,20,21,22]. The latest systematic review and meta-analysis reported that the pooled data reaching the target site, biliary cannulation, and procedural success rates were 86.6%, 90%, and 75.8%. Adverse events occurred in 6.6% of the procedures [23]. Fatal pancreatitis and intestinal perforation requiring surgical operation were included in the report. Although these were acceptable adverse event rates, we must be mindful that fatal adverse events can occur. It was also reported that bilateral stenting (partial stent-in-stent placement method) using self-expandable metallic stents for patients with hilar bile duct cancer was possible by use of short SBE [24].

**Figure 2:**
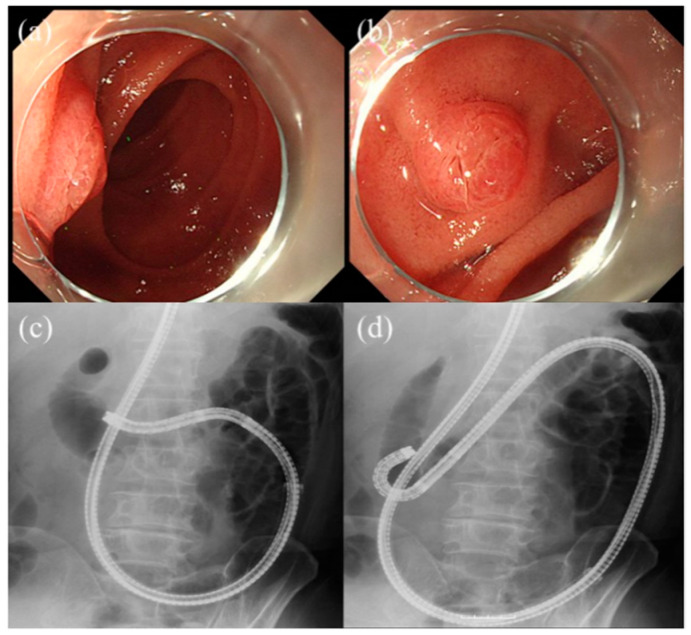
Retroflex position: (**a**,**c**). The papilla is positioned tangential, so it is difficult for biliary cannulation. (**b**,**d**) The endoscope is advanced while using the up angle at the inferior duodenal angle. As a result, it provides a better view of the papilla.

Selective biliary cannulation seems to be more difficult in patients with SAA than patients with normal anatomy. The reason is the following: the papilla appears inverted, the view of the papilla tends to be tangential, SBE is forward-viewing, and the elevator system is not equipped. There are several tips for biliary cannulation using SBE. As previously mentioned, the use of a transparent hood is effective for biliary cannulation [13]. Moreover, it was reported that suction of the papilla into the transparent cap facilitated biliary cannulation [25]. The retroflex position contributes to gaining a better view of the papilla in patients with Roux-en-Y gastrectomy. [20,26]. To achieve the retroflex position, the endoscope is advanced while using the upper angle at the inferior duodenal angle. The scope provides a J-turn form (Figure 2). Moreover, cannulation techniques, such as the double-guidewire method, insertion along the pancreatic duct (PD) stent [27], and use of the unique cannula equipped double-lumen [28] are useful.

**Figure 3:**
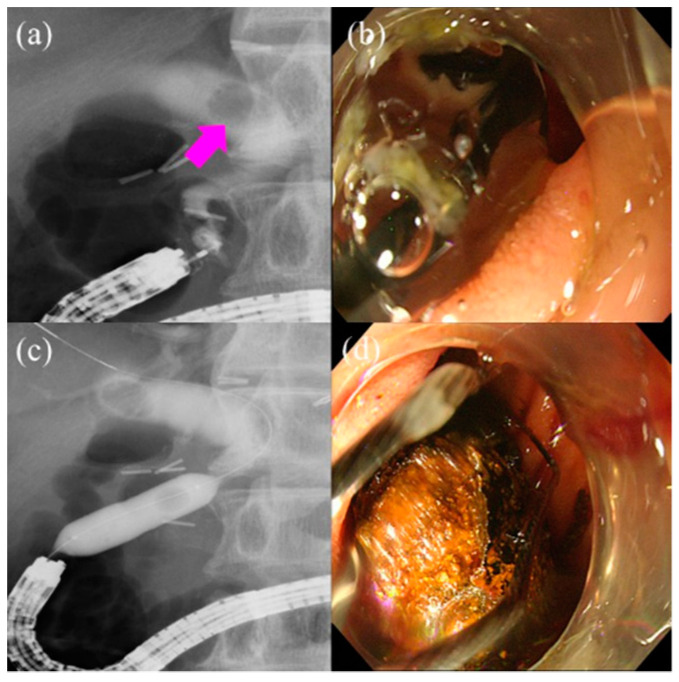
Endoscopic stone extraction using short single-balloon enteroscopy (short SBE) for patients with surgically altered anatomy (SAA): (**a**) Cholangiography showing a 15-mm biliary stone (pink arrow) in the distal bile duct. (**b**,**c**) Endoscopic papillary large balloon dilation was performed for stone extraction. The balloon was inflated up to 13-mm. (**d**) Stone extraction was completed without crushing.

Some studies have reported factors affecting procedural results. One study reported that pancreatic indications, first ERCP attempt, and no transparent hood affected procedural failure [21]. Another study reported that malignant biliary obstruction, first ERCP attempt, and Roux-en-Y reconstruction affected procedural failure [12]. Figure 3 demonstrates endoscopic stone extraction using short SBE for patients with SAA.

## 4. Double Balloon-Assisted ERCP

Table 3 shows the outcomes of DBE-assisted ERCP procedures in patients with SAA [29,30,31,32,33,34,35,36,37,38]. The latest systematic review and meta-analysis reported that the pooled data reaching the target site, biliary cannulation, and procedural success rates were 90%, 94%, and 93%. Adverse events occurred in 4% [39]. One case of intestinal perforation requiring surgery was included in the report. A single-center large cohort study reported that Billroth II gastrectomy (B-II) and the native papilla were notable risk factors for complications [40]. In that report, especially cases of B-II with an extremely short afferent loop between the gastro-jejunal anastomosis and Treitz ligament, had a risk of perforation because B-II with an extremely short afferent loop tend to receive a strong force while proceeding a scope into the afferent loop. This kind of perforation could also occur in SBE.

There are several technical tips for DBE. As previously mentioned, the retroflex position is also useful for biliary cannulation using DBE. Since the working channel port shows up in a 5:30 o’clock direction on the endoscopic screen, positioning and fixing the papilla in a 6 o’clock direction is effective to perform endoscopic sphincterotomy safely [41]. This position provides the oral protrusion and the hooding fold, which are landmarks of the direction of bile duct in performing endoscopic sphincterotomy. Furthermore, it enables confirmation whether common bile duct stones are present or not between the balloon and common bile duct during endoscopic papillary large balloon dilation [42].

Factors affecting procedural results using DBE have also been reported. One study noted that patients with surgery during childhood, biliary atresia, and second operation post-transplant were factors affecting procedure results in patients with Roux-en-Y reconstruction [36]. Another study reported that Roux-en-Y reconstruction and the first-time procedure affected the outcomes and adverse events [38]. In the report, a physician in training did not significantly affect the outcomes.

## 5. Other Device-Assisted ERCP

There are several reports of ERCP using other devices. Motorized spiral enteroscopy (PSF-1, Olympus Medical Systems, Tokyo, Japan) with a working length of 168 cm, and with a working channel diameter of 3.2 mm is available from 2015. The drive motor located in the endoscope handle is activated by foot pedals and controls the direction and speed of rotation of a coupler located in the middle of the endoscope’s insertion tube. The single-use spiral assembly is composed of corrugated tubing with an atraumatic plastic spiral bonded to its exterior. It relies on rotation of the spiral component to “pleat” or “un-pleat” the bowel either on or off the insertion tube as the spiral thread rotates in a clockwise or counterclockwise direction, respectively [43,44,45]. It has been evaluated in prospective clinical trials and shown to be safe and effective for deep enteroscopy [45]. Moreover, in view of ERCP, it allows the uses of standard ERCP-accessories in the same way as short SBE and DBE. Actually, there is one report published regarding motorized spiral enteroscopy-assisted ERCP in a patient with SAA, showing successful and rapid enteroscopic access, cannulation, and balloon dilation therapy [46]. Although further studies are needed, it could be the upcoming ERCP technology in pa-tients with SAA.

Moreover, laparoscopy-assisted ERCP (LA-ERCP) is accomplished by placing a trocar in the remnant stomach under laparoscopic guidance followed by insertion of the conventional duodenoscope through the trocar to reach the papilla. ERCP is then carried out in a standard method. The advantage of LA-ERCP is that the duodenoscope, which is used for ERCP when normal anatomy is available. It was reported that LA-ERCP achieved high success rates [47,48]. A multicenter study reported that the procedural success, and adverse events rates were 98%, and 18% (laparoscopy related, 10%, ERCP related, 7%, both, 1%) [49]. Although there is a high success rate, the overall adverse event rate was high due to the added laparoscopy-related events.

## 6. Interventional EUS

Despite the high effectiveness reported for BE-assisted ERCP in patients with SAA, it has several challenges for successful completion of procedures. Alternative treatment modalities are needed for some cases. Percutaneous transhepatic biliary drainage (PTBD) has been traditionally performed in these patients despite PTBD being associated with a higher adverse event rate than ERCP [50]. PTBD is conventionally performed using the following three-step approach: (1) external drainage with confirmation of clinical improvement, (2) stent deployment with maintenance of the external drainage tube, and (3) external drainage tube removal after the confirmation of proper drainage through the stent. Although PTBD is one of the alternatives, it may be impractical for urgent cases due to the requirement of serial dilation and track maturation [51]. Moreover, external drainage tube trouble could be caused. However, PTBD is possible to perform stone extraction effectively and safely, so we can choose PTBD as the alternatives for cases of difficult stone extraction using BE.

Recently, interventional EUS has been in the spotlight as an alternative therapy for patients with difficult ERCP, such as scope insertion and biliary cannulation. Interventional EUS may be a first-line treatment in some cases, such as malignant cases with cancer invasion of the small intestines or papilla [12].

There are several drainage methods for interventional EUS [52]. The first method is the EUS-guided hepaticogastrostomy (EUS-HGS). Generally, the left intrahepatic bile duct (B 2 or 3) is punctured to make the drainage route. After cholangiography and guidewire insertion, the fistula is dilated using a dilation device followed by the placement of a biliary stent [53]. If the stomach has been resected, such as in Roux-en-Y gastrectomy cases, a puncture is performed from the jejunal limb. The second method is EUS-guided antegrade stenting (EUS-AG). After puncture of the left intrahepatic bile duct, a guidewire is directed to the papilla or hepaticojejunal anastomosis, and the biliary stent is placed via an antegrade route [54]. Moreover, the EUS-guided rendezvous technique (EUS-RV) is also a useful alternative procedure [55]. In cases of difficult biliary cannulation using a BE, after the left intrahepatic bile duct (B2 or B3) is punctured, the guidewire is directed beyond the papilla or hepaticojejunal anastomosis. As a result, the guidewire is positioned into the duodenum or jejunum. Afterward, a scope exchange from the echoendoscope to BE is carried out. The guidewire is grasped using a forceps device and pulled into the working channel. Finally, biliary cannulation through the papilla or anastomotic site is successful.

**Figure 4:**
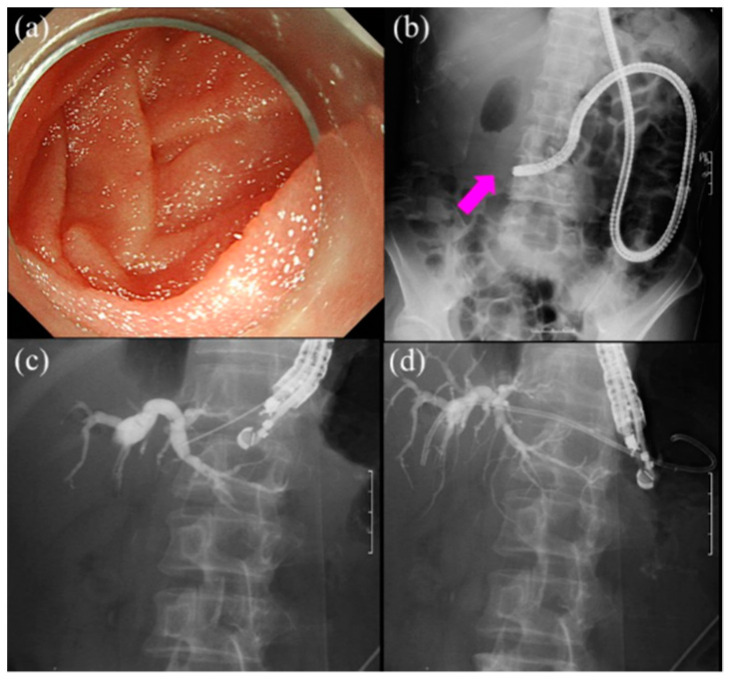
Endoscopic ultrasound-guided hepaticogastrostomy for patients with surgically altered anatomy (SAA) showing a failed case of single-balloon enteroscopy-assisted (SBE) endoscopic retrograde cholangiopancreatography (ERCP). (**a**) It was impossible to reach the papilla due to cancer invasion of the duodenum. (**b**) Fluoroscopic image showing duodenal obstruction due to cancer invasion (pink arrow). (**c**) Endoscopic ultrasound-guided hepaticogastrostomy is performed. First, B 3 is punctured using a 19-gauge needle. After puncture, we performed cholangiography to confirm the position of the guidewire. (**d**) Finally, a biliary stent was placed.

Table 4 shows outcomes of EUS-guided biliary drainage (EUS-BD) [56,57,58,59,60,61,62,63,64]. The latest systematic review and meta-analysis reported that the pooled technical success rates and clinical success rates were 91.5% and 87%, respectively. Adverse events occurred in 17.9%. The main adverse events were bile leakage (4.1%), stent migration (3.9%), and infections (3.8%) [65]. Although there were high success rates using interventional EUS, adverse events were higher than BE-assisted ERCP. Therefore, EUS-BD should be performed carefully and endoscopists should take into consideration that severe adverse events could develop. Figure 4 provides the successful EUS-HGS in a patient with SAA. Although SBE-assisted ERCP was initially performed, it failed due to cancer invasion of the small intestine.

## 7. Comparison between BE-Assisted ERCP and Interventional EUS

Some papers have conducted a comparison between BE-assisted ERCP and EUS-BD in patients with SAA. A multicenter retrospective study reported that clinical success was 88% in the EUS-BD group. It was 59.1% in the BE-assisted ERCP group (odds ratio [OR] 2.83, *p* = 0.03). The EUS-BD group completed the procedure in a shorter amount of time than the BE-assisted ERCP group (55 min vs. 95 min, *p* < 0.0001). However, adverse events occurred more often in the EUS-BD group (20% vs. 4%, *p* = 0.01) [66]. An international multicenter study compared EUS-BD and BE-assisted ERCP in patients with Roux-en-Y gastric bypass and showed that the technical success rate of EUS-BD was superior to BE-assisted ERCP (100% vs. 60%). Adverse events occurred comparably [67]. These comparison studies had lower success rates than studies in Table 2 and Table 3. These comparison studies’ population were almost all R-Y reconstruction. Studies in Table 2 and Table 3 included Billroth II gastrectomy and pancreaticoduodenectomy, which are considered to be easier than R-Y. Therefore, these success rates for BE would be lower than Table 2 and Table 3.

Although interventional EUS provided a higher success rate and shorter procedure time, adverse events tended to be high. A fatal complication, such as aberrant stent displacement into the abdominal cavity, has been reported [68]. Dedicated devices used by EUS-BD are warranted for safety. Hence, the choice between BE-assisted ERCP and interventional EUS depends on the postoperative reconstruction, patient’s condition, or the expertise of the endoscopist.

## 8. Conclusions

**Figure 5:**
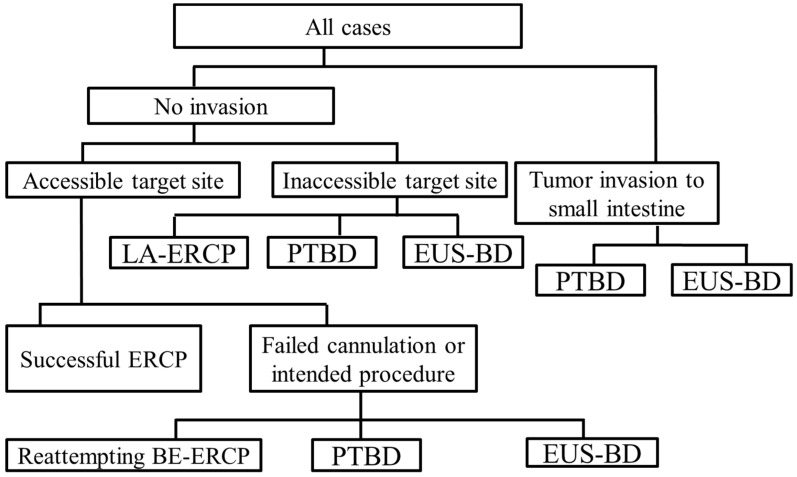
Flowchart of our proposed interventional strategy for patients with surgically altered anatomy (SAA). ERCP, endoscopic retrograde cholangiopancreatography. EUS-BD, endoscopic ultrasound-guided biliary drainage. PTBD, percutaneous transhepatic biliary drainage. LA-ERCP, laparoscopy-assisted ERCP. BE-ERCP, balloon enteroscope-assisted ERCP.

We discussed recent advances in interventional ERCP and EUS for patients with SAA. Both BE-assisted ERCP and interventional EUS have advantages and disadvantages. The choice of procedure should be individualized to the patient’s condition or the expertise of the endoscopist. We propose the following interventional strategy for patients with SAA (Figure 5). First, if tumor invasion to the small intestine can be adequately predicted prior to the procedure by cross-sectional imaging, such as computed tomography, the most appropriate technique for the case, such as PTBD or EUS-BD, can be selected as alternative interventions. During the procedure, if the target site (papilla or hepaticojejunal anastomosis) cannot be reached using a BE, laparoscopy-assisted ERCP, PTBD, or EUS-BD will be required to complete the treatment procedure. In case of failed biliary cannulation or an intended procedure, reattempting BE-assisted ERCP, PTBD, or EUS-BD should be selected according to the previous treatment.

Further improvement of both BE-assisted ERCP and interventional EUS are needed to perform effective and safe procedures for patients with SAA.

## Figures and Tables

**Table 1:** Specifications of single-balloon enteroscopy (SBE) and double-balloon enteroscopy (DBE).

Company	Olympus	Olympus	Fujifilm	Fujifilm
	SIF-Q260	SIF-H290S	EN-580T	EI-580BT
Angle of view	140°	140°	140°	140°
Outer diameter (mm)	9.2	9.2	9.4	9.4
Working length (mm)	2000	1520	2000	1550
Working channel diameter (mm)	2.8	3.2	3.2	3.2
Passive bending	No	Yes	No	No
High-force transmission	No	Yes	No	No
The adaptive bending	No	No	No	Yes
Advanced force transmission	No	No	No	Yes

SBE, single-balloon enteroscopy. DBE, double-balloon enteroscopy.

**Table 2:** Outcomes of single balloon enteroscopy (SBE)-assisted endosopic retrograde cholangiopancreatography (ERCP) procedure in patients with surgically altered anatomy (SAA).

Authors	Year	Reaching the Target Site Success, % (*n*)	Biliary Cannulation Success, % (*n*)	Procedural Success, % (*n*)
Wang et al. [15]	2010	81.3 (13/16)	92.3 (12/13)	75.0 (12/16)
Shah et al. [16]	2013	68.9 (31/45)	87.1 (27/31)	60.0 (27/45)
Lenze et al. [17]	2014	73.1 (19/26)	78.9 (15/19)	57.7 (15/26)
Trindade et al. [13]	2015	87.5 (49/56)	89.8 (44/49)	71.4 (40/56)
Kawamura et al. [18]	2015	88.9 (24/27)	83.3 (20/24)	70.4 (19/27)
Yamauchi et al. [19]	2015	90.5 (76/84)	89.5 (68/76)	77.4 (65/84)
Ishii et al. [20]	2016	91.9 (113/123)	94.1 (95/101)	88.1 (96/109)
Yane et al. [21]	2017	92.6 (188/203)	N/A	81.8 (166/203)
Tanisaka et al. [12]	2019	94.8 (181/191)	92.3 (167/181)	85.9 (164/191)
Sawas et al. [22]	2020	86.0 (37/43)	83.8 (31/37)	69.8 (30/43)

SBE, single-balloon enteroscopy. ERCP, endoscopic retrograde cholangiopancreatography. SAA, surgically altered anatomy. N/A, not available.

**Table 3:** Outcomes of double balloon endoscopy (DBE)-assisted ERCP procedure in patients with surgically altered anatomy (SAA).

Authors	Year	Reaching the Target Site Success, % (*n*)	Biliary Cannulation Success, % (*n*)	Procedural Success, % (*n*)
Aabakken et al. [29]	2007	94.4 (17/18)	88.2 (15/17)	83.3 (15/18)
Emmett et al. [30]	2007	85.0 (17/20)	94.1 (16/17)	80.0 (16/20)
Shimatani et al. [31]	2009	97.1 (100/103)	98.0 (98/100)	95.1 (98/103)
Cho et al. [32]	2011	86.2 (25/29)	96.0 (24/25)	82.8 (24/29)
Tsutsumi et al. [33]	2015	98.6 (71/72)	100 (71/71)	98.6 (71/72)
Cheng et al. [34]	2015	94.8 (73/77)	94.5 (69/73)	87.0 (67/77)
Shimatani et al. [35]	2016	97.7 (304/311)	96.4 (293/304)	92.3 (287/311)
Liu et al. [36]	2017	75.6 (65/86)	92.3 (60/65)	69.8 (60/86)
Kashani et al. [37]	2018	93.8 (121/129)	N/A	88.4 (114/129)
Uchida et al. [38]	2020	94.3 (759/805)	N/A	90.7 (730/805)

DBE, double-balloon enteroscopy. ERCP, endoscopic retrograde cholangiopancreatography. SAA, surgically altered anatomy. N/A, not available.

**Table 4:** Outcomes of endoscopic ultrasound (EUS)-guided biliary drainage.

Authors	Year	Technical Success, % (*n*)	Clinical Success, % (*n*)	Adverse Events, % (*n*)
Shah et al. [56]	2011	70.5 (62/88)	70.5 (62/88)	6.8 (6/88)
Khashab et al. [57]	2013	94.3 (33/35)	91.4 (32/35)	11.4 (4/35)
Park et al. [58]	2013	91.1 (41/45)	86.7 (39/45)	8.9 (4/45)
Kawakubo et al. [59]	2014	95.3 (61/64)	N/A	18.8 (12/64)
Gupta et al. [60]	2014	88.5 (207/234)	N/A	34.6 (81/234)
Dhir et al. [61]	2015	93.3 (97/104)	89.4 (93/104)	8.7 (9/104)
Kahaleh et al. [62]	2016	91.4 (32/35)	88.6 (31/35)	25.7 (9/35)
Tsuchiya et al. [63]	2018	100 (19/19)	94.7 (18/19)	36.8 (7/19)
Minaga et al. [64]	2019	85.2 (46/54)	85.2 (46/54)	18.5 (10/54)

EUS, endoscopic ultrasound. N/A, not available.

## Data Availability

Data sharing not applicable.
